# Implementation of chronic illness care in German primary care practices – how do multimorbid older patients view routine care? A cross-sectional study using multilevel hierarchical modeling

**DOI:** 10.1186/1472-6963-14-336

**Published:** 2014-08-07

**Authors:** Juliana J Petersen, Michael A Paulitsch, Karola Mergenthal, Jochen Gensichen, Heike Hansen, Siegfried Weyerer, Steffi G Riedel-Heller, Angela Fuchs, Wolfgang Maier, Horst Bickel, Hans-Helmut König, Birgitt Wiese, Hendrik van den Bussche, Martin Scherer, Anne Dahlhaus

**Affiliations:** 1 Institute of General Practice, Goethe-University Frankfurt am Main, Theodor-Stern-Kai 7, Frankfurt/Main 60590, Germany; 2 Institute of General Practice and Family Medicine, Jena University Hospital, Friedrich Schiller University, Bachstraße 18, Jena 07743, Germany; 3 Department of Primary Medical Care, University Medical Center Hamburg-Eppendorf, Martinistraße 52, Hamburg 20246, Germany; 4 Central Institute of Mental Health, Medical Faculty Mannheim/Heidelberg University, J5, Mannheim 68159, Germany; 5 Institute of Social Medicine, Occupational Health and Public Health, University of Leipzig, Philipp-Rosenthal-Straße 55, Leipzig 04103, Germany; 6 Institute of General Practice, University of Düsseldorf, Moorenstraße 5, Düsseldorf 40225, Germany; 7 Department of Psychiatry and Psychotherapy, University of Bonn, Sigmund-Freud-Straße 25, Bonn 53105, Germany; 8 Department of Psychiatry, Technical University of Munich, Ismaninger Str. 22, München 81675, Germany; 9 Department of Health Economics and Health Services Research, University Medical Center Hamburg-Eppendorf, Martinistr. 52, Hamburg 20246, Germany; 10 Working Group Medical Statistics and IT-Infrastructure, Institute of General Practice, Hannover Medical School, Hannover 30625, Germany

**Keywords:** Chronic disease, Multimorbidity, Chronic care model, Assessment of care, Patient-centred care

## Abstract

**Background:**

In primary care, patients with multiple chronic conditions are the rule rather than the exception. The Chronic Care Model (CCM) is an evidence-based framework for improving chronic illness care, but little is known about the extent to which it has been implemented in routine primary care. The aim of this study was to describe how multimorbid older patients assess the routine chronic care they receive in primary care practices in Germany, and to explore the extent to which factors at both the practice and patient level determine their views.

**Methods:**

This cross-sectional study used baseline data from an observational cohort study involving 158 general practitioners (GP) and 3189 multimorbid patients. Standardized questionnaires were employed to collect data, and the Patient Assessment of Chronic Illness Care (PACIC) questionnaire used to assess the quality of care received. Multilevel hierarchical modeling was used to identify any existing association between the dependent variable, PACIC, and independent variables at the patient level (socio-economic factors, weighted count of chronic conditions, instrumental activities of daily living, health-related quality of life, graded chronic pain, no. of contacts with GP, existence of a disease management program (DMP) disease, self-efficacy, and social support) and the practice level (age and sex of GP, years in current practice, size and type of practice).

**Results:**

The overall mean PACIC score was 2.4 (SD 0.8), with the mean subscale scores ranging from 2.0 (SD 1.0, subscale goal setting/tailoring) to 3.5 (SD 0.7, delivery system design). At the patient level, higher PACIC scores were associated with a DMP disease, more frequent GP contacts, higher social support, and higher autonomy of past occupation. At the practice level, solo practices were associated with higher PACIC values than other types of practice.

**Conclusions:**

This study shows that from the perspective of multimorbid patients receiving care in German primary care practices, the implementation of structured care and counseling could be improved, particularly by helping patients set specific goals, coordinating care, and arranging follow-up contacts. Studies evaluating chronic care should take into consideration that a patient’s assessment is associated not only with practice-level factors, but also with individual, patient-level factors.

**Trial registration:**

Current Controlled Trials ISRCTN89818205.

## Background

In primary care, patients with multiple chronic conditions are the rule rather than the exception [1-3]. Compared to patients with single conditions, multimorbid patients are more likely to die prematurely, to be admitted to hospital and to have poorer quality of life [4-6]. Although evidence exists that a structured, proactive and patient-centered approach helps to improve health outcomes, current delivery of care is often fragmented and event-driven [7]. One widely accepted evidence-based framework for improving chronic care is the Chronic Care Model [8,9]. The CCM supports the provision of high-quality care and emphasizes the importance of continuity of care in a strong primary care sector. It aims to ensure care is planned, proactive and patient-centered, rather than reactive and focused on acute episodes, and it is designed to improve care in health systems at the community, organization, practice and patient levels. The model identifies key elements as essential to the provision of high-quality care to patients with chronic illnesses, i.e. self-management support, provision of clinical information systems, delivery system redesign, decision support, improved health care organization, and the use of community resources. For instance, the element “delivery system redesign” focuses on transforming a system that is essentially reactive into one that is proactive, thus ensuring the patient receives structured and planned care, as well as follow-up consultations, as part of a standard procedure [10]. Interventions involving one or more elements of the CCM have shown beneficial effects on clinical outcomes and care processes [11-13]. In Germany, little is known about the degree of implementation of elements of the CCM in routine primary care for multimorbid patients. The ‘Patient Assessment of Chronic Illness Care’ (PACIC) questionnaire can be used to assess patients’ perceptions in this respect [14]. The PACIC has been used increasingly in primary care research in recent years [15]. In an ongoing cluster-randomized controlled trial, for example, Van Lieshout et al. are using the PACIC to analyze the effectiveness of a tailored implementation program for patients with chronic heart failure in general practices [16]. The PACIC questionnaire has been translated and validated in several languages, including German [17] and Dutch [18]. In Germany, the PACIC has also been used to evaluate disease management programs [19] that have been implemented in primary care nationwide and are aimed at promoting evidence-based chronic care that contains similar core elements to those used in the CCM [7]. When interpreting study findings based on the PACIC, it is important to understand what factors influence a patient’s assessment of care. Previous research indicates that a patient’s assessment of chronic illness care may depend, not only on the care received, but also on the patient him or herself. Cramm and Nieboer investigated patients with cardiovascular diseases and chronic obstructive pulmonary disease in the Netherlands and found that younger and less depressed patients report higher PACIC scores [20]. In a large integrated health care delivery system, Glasgow et al., who developed the PACIC, found a slight correlation between PACIC values and age and gender of the treated patients, while education was not associated with them [14]. In a sample of patients with osteoarthritis, on the other hand, Rosemann and colleagues found that female gender and age were weakly positively correlated with PACIC sum scores, while education was slightly negatively correlated with them. However, none of these correlations were statistically significant [17]. Ludt et al., who analyzed a sample of patients with coronary heart disease receiving structured chronic care in a number of European countries, found that at the patient level, male gender, more frequent practice attendance, and fewer conditions, are associated with higher PACIC scores [21].

The aim of this study was to describe how multimorbid older patients assess the routine chronic care they receive in primary care practices in Germany, and to simultaneously explore the extent to which practice- and patient-level factors determine patients’ assessments.

## Methods

### Study design

This cross-sectional study used baseline data from a prospective cohort study of multimorbid older patients receiving care in primary care practices. The study design and recruitment procedures have already been published in detail [22]. In brief, patients were randomly selected and recruited from 158 general practices in eight German cities. The inclusion criteria were multimorbidity (defined as three or more different diagnoses of chronic diseases), aged between 65 and 84 years, and at least one visit to the general practitioner within the last three months. The following criteria led to exclusion: 1) resident of a nursing home, 2) participation in other research studies, 3) not known well enough by their general practitioner, 4) life expectancy of less than three months, 5) inadequate knowledge of German, 6) insufficient ability to participate in interviews (e.g. blindness, deafness) or 7) unable to give consent (e.g. demented patients). All participants gave informed consent, and the study was approved by the ethics committees of all participating centers [22].

### Data collection and assessment procedure

Data collection took place between July 2008 and October 2009. Participating patients provided comprehensive self-reported data on their socio-economic, health and functional status in standardized questionnaires. Additional clinical information was obtained from the GPs. The dependent variable was assessed using the Patient Assessment of Chronic Illness Care instrument [14]. The PACIC questionnaire contains 20 items on five subscales that are based on conceptual categories of the CCM, i.e. patient activation (3 items), delivery system design/decision support (3 items), goal setting/tailoring (5 items), problem solving/contextual counseling (4 items) and follow-up/coordination (5 items). Each item is scored on a five-point Likert scale that ranges from 1 (‘almost never’) to 5 (‘almost always’), with higher scores indicating better patient-perceived quality of chronic illness care. Patients were asked to assess the care provided by their GP rather than by the team as a whole. Socio-demographic variables considered in the analyses at the patient-level included the following: age, sex, education level (operationalized using the Comparative Analysis of Social Mobility in Industrial Nations (CASMIN) classification into low, middle and high education level) [23], past occupation (grouped according to degree of autonomy at work) [24], and regular monthly net income, adjusted for household size. Morbidity of patients was assessed using a standardized questionnaire for GPs which covers a list of 46 chronic conditions [25,26]. The severity of each chronic condition was assessed by the GP on a 5-point Likert scale (0 ‘marginal’, 1 ‘low’, 2 ‘medium’, 3 ‘severe’ and 4 ‘very severe’). The weighted disease count was then calculated by summing up the severity ratings. Since the cumulative effect of the diseases may not provide an accurate characterization of the level of multimorbidity [27], we also included activities of daily living as a measure of functional impairment (Instrumental Activities of Daily Living (IADL) scale) [28], health-related quality of life (visual analogue scale) as a measure of self-rated health [29], and graded chronic pain as a combined measure of pain intensity and pain-related disability (Graded Chronic Pain Scale (GCPS)) [30]. Furthermore, on the basis of evidence from previous research indicating that they are associated with a patient’s assessment of chronic illness care, we included depression (Geriatric depression scale, GDS) [31], frequency of practice attendance, and existence of a DMP disease [17,20,21]. We also included self-efficacy (self-efficacy scale) [32], and social support, e.g. by family members and neighbors (F-SozU K-14 scale) [33], as independent variables because these factors may also influence satisfaction with care or use of health services [34,35]. We hypothesized that higher social support and higher self-efficacy would be associated with a better assessment of chronic care by patients. Practice variables considered in the analyses were age and sex of GP, years in current practice, practice size (operationalized as number of patients treated in the practice over the previous 3 months) and type of practice (solo practice, shared practice (i.e. GPs shared the practice with other physicians, but cared for their regular patients themselves), and group practice (i.e. practice and patients were shared with other physicians).

### Statistical analysis

We calculated mean overall PACIC and mean subscale scores by averaging them across the corresponding items [14]. Due to the multilevel structure of the data, we calculated a multilevel hierarchical linear model, taking into account patient observations (level 1), nested within general practices (level 2). We constructed multilevel models in several steps. In a first step we calculated a ‘null’ model with no predictor variables to test whether the mean overall PACIC scores varied significantly across the sample (random-effect with a *p*-value < 0.05), and assessed the Intraclass Correlation Coefficient (ICC) to quantify similarity within the groups. In a second step, fixed level 1 and level 2 variables were added and tested for their association with the dependent variable, the PACIC score. We report regression coefficients and their confidence intervals, as well as *p*-values. All *p* values were 2-sided and the chosen significance level was 0.05. In both models, estimates were calculated by means of Restricted Maximum Likelihood.

Income data was missing in 12.2% of cases. As a result of the missing data, we could not calculate the patient assessment of chronic illness care sum score in 13.1%, the weighted count of chronic conditions in 4.8%, and the Graded Chronic Pain Scale in 1.7% of cases. The percentage of missing values did not exceed 0.6% in any other categories. Missing values were imputed using the hot deck method, in which missing values are replaced using observed values from a responding unit that is as similar as possible to the non-responding one [36]. Imputation of missing values was performed using the R 2.13.0 package StatMatch [37]. Further details concerning the applied imputation method have been published elsewhere [26]. Statistical analyses were carried out using HLM 7.0 [38] for multilevel analyses and SPSS Statistics 19 [39] for other analyses.

## Results

### Practice and patient characteristics

The final sample consisted of 158 general practitioners and 3189 multimorbid patients (45.2% of 7044 eligible patients). The socio-demographic characteristics of the sample and a comparison of participating with non-participating patients have been described in detail elsewhere [26]. In brief, the mean age of the GPs was 50.2 (SD 7.7) years, and 60.8% were male. The GPs had owned their practices for an average of 15 years, and 81.0% worked in solo or two-physician practices. More than half (52.5%) of the GPs were solo practitioners, 12.7% shared their practice with other physicians (yet cared for their regular patients on their own), and 34.8% worked in a group practice (i.e. they shared practice and patients with other physicians, usually GPs). In 51.3% of the practices, 1000 or more patients had been treated in the previous three months, and in 48.7% of the practices, fewer than 1000 patients had been treated in the previous three months. The characteristics of the patient sample are displayed in Table 1. The mean number of patients included per practice was 20 (SD 8.1).

**Table 1:** Socio-demographic characteristics of the patient sample

**Characteristics**^**a**^	
Mean age (yrs), mean (SD)	74.4 (5.2)
Sex, n (%)	
Female	1891 (59.3)
Male	1298 (40.7)
Education (in CASMIN grades), n (%)	
Grade 1 (low)	1986 (62.3)
Grade 2 (medium)	856 (26.8)
Grade 3 (high)	347 (10.9)
Five most prevalent conditions in sample, n (%)	
Hypertension	2483 (77.9)
Lipid metabolism disorders	1867 (58.5)
Chronic low back pain	1577 (49.5)
Joint arthrosis	1382 (43.3)
Diabetes mellitus	1199 (37.6)
Number of chronic conditions, mean (SD)	7.0 (2.5)
Weighted count of chronic conditions, mean (SD)^b^	11.3 (5.1)

^a^These analyses are based on n = 3189 patients; missing PACIC sum scores were imputed.

^b^based on a list of 46 chronic conditions.

### Distributions of PACIC scores

Table 2 displays descriptive statistics for scores on the PACIC scales. The mean overall score was 2.4 (SD 0.8), the mean scores for the subscales ranged from 2.0 (subscale goal setting/tailoring) to 3.5 (delivery system design/decision support).

**Table 2:** **Score distributions of the PACIC**^**a**^

	**Mean**	**SD**
Overall PACIC score^b^	2.4	0.8
Patient activation	2.6	1.2
Delivery system design/decision support	3.5	0.7
Goal setting/tailoring	2.0	1.0
Problem solving/contextual	2.5	1.1
Follow-up/coordination	2.1	0.9

^**a**^Each PACIC item is scored on a five-point Likert scale that ranges from 1 (‘almost never’) to 5 (‘almost always’), with higher scores indicating better patient-perceived quality of chronic illness care.

^b^These analyses are based on n = 3189 patients; missing PACIC sum scores were imputed.

### Association between PACIC and potential explanatory variables

**Figure 1:**
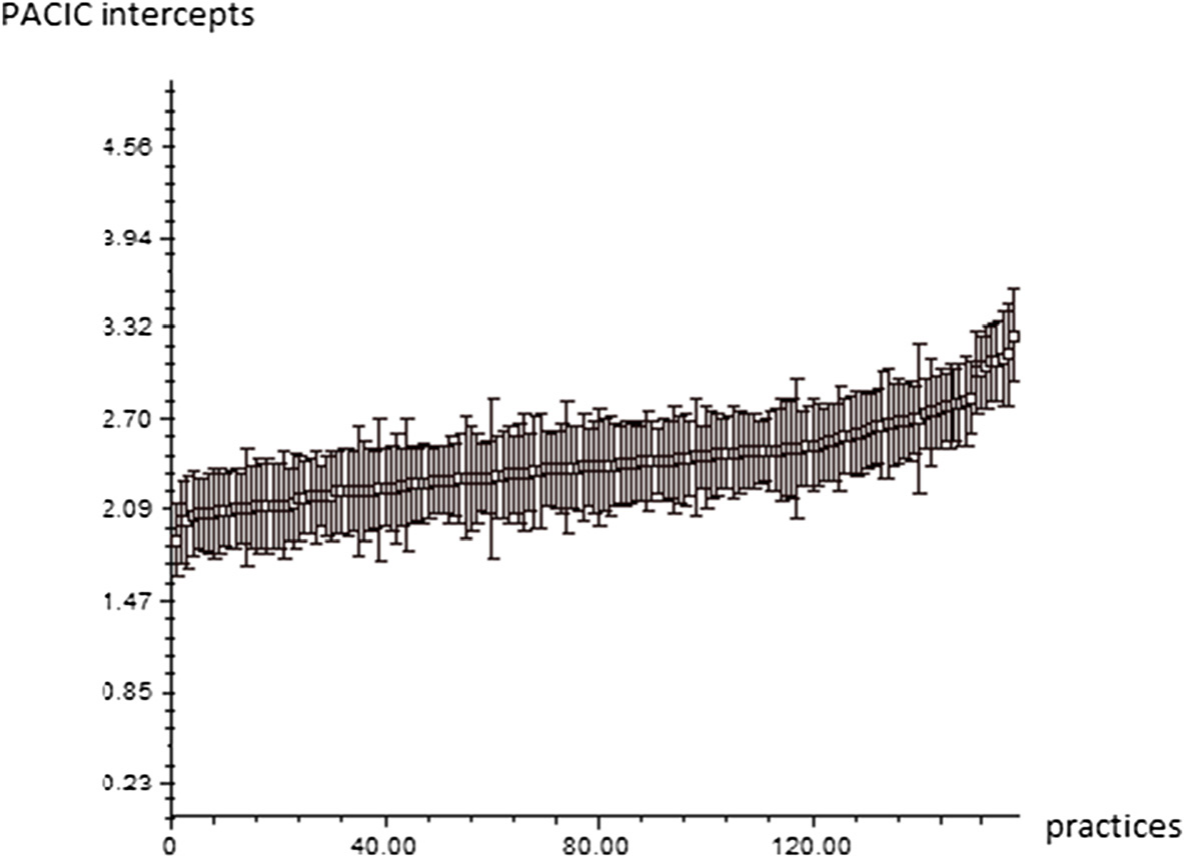
**PACIC intercepts in null model.** (i.e., mean overall PACIC scores per practice; n=3189 patients and n=158 practices).

The results of the ‘null’ model with no predictor variables indicate that the overall mean PACIC scores (intercepts) varied statistically significantly (random-effect with a p-value < 0.05) between practices, and thus required multilevel analysis. Figure 1 displays the mean scores for each practice (intercepts). The ICC was 12.9%, i.e. 12.9% of the total variance occurred at the practice level.

Table 3 displays the results of the final multilevel hierarchical model with the overall mean PACIC scores as the dependent variable. At the patient level, higher PACIC scores were associated with the existence of at least one DMP disease. The coefficient of this association was 0.11., i.e. having a DMP disease was associated with a 0.11 point higher PACIC score compared to not having it. PACIC scores were also statistically significantly associated with more frequent contacts with the GP (coefficient 0.01), higher social support (0.16), higher autonomy in the patient’s past occupation (0.04), and higher pain disability (0.05). At the practice level, sharing/group practices were statistically significantly associated with lower mean PACIC scores (coefficient −0.13) compared to single practices.

**Table 3:** **Association between potential explanatory variables and overall patient assessment of chronic illness care (PACIC)**^a^

**Variables**	**Regression coefficient**	**95% confidence interval**	***p*** **value**^**b**^
**Patient characteristics**			
**Sex**			
Female	−0.08	−0.2; 0.04	0.165
Male	Reference		
**Age (min. 65, max. 84 years)**			
Continuous	−0.00	−0.00; 0.00	0.23
**Education level (CASMIN classification: 1 = low, 2 = middle, 3 = high)**			
Continuous	0.03	−0.01; 0.07	0.22
**Autonomy of former occupation (value range from 0 = no occupation to 5 = occupation with high autonomy)**			
Continuous	0.04	0.02; 0.06	**0.005**
**Household-adjusted regular monthly net income (in €)**			
Continuous	−0.00	−0.00; 0.00	0.23
**No. of contacts with GP**			
Continuous	0.01	−0.01; 0.03	**0.006**
**Weighted count of chronic conditions**			
Continuous	0.01	0.01; 0.01	0.095
**Existence of a DMP disease**^**c**^			
Yes	0.11	0.05; 0.17	**<0.001**
No	Reference		
**Depression (GDS, possible values range: 0–15)**			
Continuous	−0.00	−0,02; 0,02	0.70
**Instrumental activities of daily living (IADL, possible value range: 0–8)**			
Continuous	−0.01	−0.05; 0.03	0.67
**Quality of life (visual analogue scale, possible value range: 0–100)**			
Continuous	0.00	0.00; 0.00	**0.03**
**Graded Chronic Pain Scale (GCPS grades: 0 = pain free to 4 = high disability, severely limiting)**			
Continuous	0.05	0.03; 0.07	**<0.001**
**Self-efficacy scale (possible mean score range: 1–4)**			
Continuous	0.04	−0.02; 0.1	0.25
**Social support (F-SozU K-14; possible mean overall score range: 1–5)**			
Continuous	0.16	0.12; 0.20	**<0.001**
**GP characteristics**			
**Sex**			
Female	0.12	0.00; 0.24	**0.044**
Male	Reference		
**Years of ownership of the practice**			
Continuous	−0.01	−0.01; −0.01	0.067
**Practice type**			
Shared or group practice	−0.13	−0.23; −0.03	**0.016**
Solo practice	Reference		
**Practice size, i.e. number of patients treated in last three months**			
Continuous	−0.01	−0.07; 0.05	0.77

^a^These analyses are based on n = 3189 patients; missing values were imputed

^b^*p* values marked in bold are statistically significant on the basis of a significance level of 0.05.

^c^German DMPs exist for the following diseases: breast cancer, diabetes mellitus type I or II, coronary heart disease, chronic obstructive pulmonary disease, bronchial asthma.

Due to the high percentage of missing values in the overall mean PACIC score (13.1%), we also calculated a multilevel hierarchical model that only included patients with complete overall mean PACIC scores. We found very similar results to those calculated using imputed data, with the following two exceptions: The autonomy of former occupation – which was statistically significantly associated with PACIC in the model that used imputed data – was no longer statistically significantly associated with the PACIC scores in the model that only included patients whose PACIC scores were complete (regression coefficient 0.03 [CI −0.01;0.07]; p = 0.092). The weighted count of chronic conditions – which was not statistically significantly associated with PACIC scores in the calculations based on imputed data – became statistically significantly associated with PACIC scores when we only included patients whose PACIC scores were complete (regression coefficient 0.01 [CI 0.01;0.01]; p = 0.041).

## Discussion

This study shows that from the perspective of multimorbid patients elements of chronic care have not yet been fully implemented in primary care practices in Germany. While some key elements, such as delivery system redesign/decision support (e.g. ‘Satisfied that my care was well organized’) have been well implemented in routine care, other elements such as helping the patient to set specific goals and arranging follow-ups are less common. Patients’ assessment of care was not only associated with practice- but also with patient-level factors.

The mean PACIC score of 2.4 reported in this study is one of the lowest described to date. This probably reflects a “real life scenario” with respect to the care that multimorbid patients actually receive in Germany, as it is generally criticized for being fragmented and event-driven [7,9,40]. Wensing et al. reported a similar mean score (2.3) in patients with COPD that had been treated in rural general practices in the Netherlands, but many other studies showed higher scores, with mean scores of 2.7 or 2.8 in samples of patients with cardiovascular disease [21,41], and scores of above 3 in patients with depression [42]. The difference is probably attributable to the fact that most previous research has focused on patients that are enrolled in structured programs/DMPs (e.g. [21]), or on patients receiving specific kinds of chronic care, such as case management (e.g. [42]). Another possible explanation is that the patients in our sample presented greater disease severity due to their multiple chronic conditions. Delivering high-quality care to these patients may be difficult due to their complex needs [43]. The finding that mean scores were highest for the subscale delivery system redesign/decision support and lowest for goal setting/tailoring is in line with recent findings in primary health care research, which have indicated a similar pattern in several other European countries [20,21], as well as in Canada [44]. We found that only 12.9% of the total variance occurred at a practice level, and that most of the variance was due to differences between patients and random error. This would appear to be a general limitation in questionnaires on satisfaction or experience of care that we think deserves more attention in studies that use the PACIC. Salisbury et al. have shown that specific measures of a patient’s experience reveal differences between practices more clearly than do conventional measures of satisfaction [45]. This is probably also true of the PACIC – in fact, the designers of the instrument have incorporated conventional questions relating to satisfaction (e.g. “satisfied that my care was well organized”) besides ratings involving specific events (e.g. “given a copy of my treatment plan”).

On the practice level, solo practices in this study were associated with higher PACIC scores than group or shared practices. This may indicate that smaller organizational units promote a stronger link between patients and providers. Lévesque et al., who analyzed the influence of different organizational models of primary health care on the PACIC, found that patients of solo practitioners and family medicine groups rated their care more highly than patients of specialist clinics [44]. At the patient level, higher PACIC values were associated with having a DMP disease. This finding was to be expected, since, similar to CCMs, DMPs combine many elements of structured care. Szecsenyi et al. have shown that patients registered in the DMP for diabetes view their care as more structured than those who are not [19]. It should be taken into account, however, that we had no information on whether an individual patient, who had a disease for which a DMP program was available, had actually enrolled in one. This may have led to an underestimate of the effect. However, since DMPs have been implemented successfully in Germany for more than 10 years now, and 6 million patients have enrolled in them, we can assume that practices nowadays provide more structured care to all eligible patients than they used to. Similar to Ludt et al. [21], we found that higher PACIC values were also associated with more frequent GP contacts. In a systematic review of patient-physician relationships, Ridd et al. showed that regular contact to the practice appeared to be one of the main criteria determining how patients experience the care they receive, and that patients with long-term and complex problems prefer to consult a single doctor [46]. Contrary to Cramm et al., [20], we did not find an association between PACIC scores and either age, or depressive symptoms. This dissimilarity is probably attributable to differences in the analyzed samples: Our sample consisted of multimorbid patients with a mean age of 74 (SD 5) years, whereas Cramm et al. studied patients with cardiovascular disease and a mean age of 64 (SD 10) years, as well as patients with chronic obstructive pulmonary disease and a mean age of 66 (SD 11) years [20]. The smaller standard deviation of age distribution in our sample is due to our inclusion criteria (age 65–84) and explains why our sample was more homogeneous in terms of age distribution than the sample of Cramm et al. This makes it more difficult to ascertain whether age could be a potential explanatory variable. One noteworthy and to our knowledge new finding in this study was that higher PACIC scores were associated with higher social support and higher autonomy in the patient’s past occupation. Muller et al. showed that patients with complex jobs that offer considerable autonomy are likely to have better health status [47]. A study in Scotland found that people living in the most deprived areas are more likely to develop multimorbidity at a young age and to have greater mental health problems [48]. The finding in this study that a patient’s characteristics influence how he or she experiences received chronic care raises the question whether this reflects different expectations, different ways of answering study questions or differences in the care provided to different types of patients within the same practices. This is an important subject and should be considered in further studies on quality of care [45]. A recent review on strategies to improve health outcomes in multimorbid patients in primary care indicates that most studies fail to adequately consider the impact of socio-economic factors, and highlights the importance of considering the possible differential effects of interventions in different socio-economic groups [49]. In this study, the percentage of practices with one or two physicians was 81%, which is typical for the setting. Such practices tend to work independently of each other and GPs often own the practice in which they work. In this setting, financial and personnel resources are often limited, and not comparable to academic or integrated settings in health maintenance organizations, where elements of the CCM are easier to implement. Over recent years, innovative ways of introducing elements of the CCM into German primary care practices have been investigated. One promising method is the involvement of specially trained healthcare assistants in the care of patients with chronic conditions [3,50,51]. Healthcare assistants are employed in the majority of German primary care practices and are less qualified than nurses. Research has indicated that trained healthcare assistants can successfully work as case managers and perform interventions designed in accordance with the CCM, e.g. for patients with depression [52] and patients with osteoarthritis [53]. An ongoing study by Freund et al. will answer the question whether a complex, multifaceted intervention involving healthcare assistants as case managers reduces the likelihood of the (re-)hospitalization of multimorbid patients [54]. Besides CCM-based interventions, other organizational or patient-oriented complex interventions were also shown to be effective in improving health outcomes among multimorbid patients in primary care [49].

### Strengths and limitations

The major strength of this study is that the prospective cohort study from which we took the baseline data is one of the largest studies on the characteristics and care of multimorbid patients in primary care in Germany. A comparison of non-participants with participants indicated that in terms of sex and the most common chronic conditions, the sample in this study was roughly representative of multimorbid older patients in primary care practices in Germany. Participants were, however, slightly younger than non-participants [26]. The cross-sectional study design means we cannot draw causality assumptions, and this is a clear limitation when interpreting the results. A further limitation is that we could not calculate the overall mean PACIC score for 13.1% of all patients due to missing values. This proportion is relatively high and probably attributable to the fact that our sample of multimorbid older patients had problems understanding some of the questions. The problem of missing PACIC data has already been pointed out by Wensing et al., who reported that 22% to 35% of patients with diabetes or COPD treated in rural practices in the Netherlands did not provide answers to specific items in the PACIC [18]. A comparison of the multilevel hierarchical modeling results (including cases with imputed data vs. cases with complete data) was reassuring in this regard, since the majority of findings remained very similar. Some uncertainty remains, however, in the interpretation of the association between PACIC and both autonomy of former occupation and weighted count of chronic conditions, since the results of the analyses with imputed data were different from those with non-imputed data in these two cases. Furthermore, the psychometric properties of the PACIC are the subject of ongoing discussion. While there is no doubt that the instrument itself is up to the task of assessing whether care is provided in accordance with the CCM [15], major criticisms concern the ways in which factorial validity and internal consistency are evaluated [15,55]. Patients may also be reluctant to criticize their GP, especially if they have a good, long-term relationship with him or her [46]. There is no single standardized way of measuring quality of care. In this study we have assessed the care received by measuring patients’ views. Since previous research has shown that patient satisfaction reports or evaluations of their healthcare experiences are not necessarily consistent with other measures of quality [56], we do not assume that the inclusion of quality indicators would have strongly influenced our results.

## Conclusions

This study shows that from the perspective of multimorbid patients, the implementation of structured chronic care and counseling could be improved in primary care practices in Germany, particularly with respect to helping patients set specific goals and arranging follow-up contacts. Studies evaluating the care of multimorbid patients should adequately take into consideration that a patient’s assessment is associated not only with practice-level factors, but also with individual, patient-level factors.

## Abbreviations

CASMIN: Comparative analysis of social mobility in industrial nations; CCM: Chronic care model; DMP: Disease management program; GCPS: Graded chronic pain scale; GP: General practitioner; IADL: Instrumental activities of daily living; ICC: Intraclass correlation coefficient; PACIC: Patient assessment of chronic illness care.

## Competing interests

The authors declare that they have no competing interests.

## Authors’ contributions

JJP, MAP and AD participated in the conception and data collection of the MultiCare study, performed the statistical analyses and drafted the manuscript. KM, JG, HH, SW, SRH, AF, WM, HB, HHK, BW, HVB and MS participated in the conception, design and data collection of the MultiCare study, and revised the manuscript critically for important intellectual content. All authors read and approved the final manuscript.

## Authors’ information

Hendrik van den Bussche and Martin Scherer: Principal investigators.

## Pre-publication history

The pre-publication history for this paper can be accessed here:

http://www.biomedcentral.com/1472-6963/14/336/prepub
